# Phase Behavior and Phase Diagram of Polystyrene-*b*-Poly(Perfluorooctylethyl Acrylates)

**DOI:** 10.3390/polym12040819

**Published:** 2020-04-04

**Authors:** Yu Shao, Hui Dai, Meng Zhao, Bin Li, Jianan Yao, Wen-Bin Zhang, Hui Li

**Affiliations:** 1 Center for Advanced Low-Dimension Materials, Donghua University, Shanghai 201620, China; boukephalas@outlook.com (Y.S.); bli@dhu.edu.cn (B.L.); yjn@dhu.edu.cn (J.Y.); 2 Beijing National Laboratory for Molecular Sciences, Key Laboratory of Polymer Chemistry and Physics of Ministry of Education, Center for Soft Matter Science and Engineering, College of Chemistry and Molecular Engineering, Peking University, Beijing 100871, China; wenbin@pku.edu.cn; 3 School of Materials Science and Engineering, East China University of Science and Technology, Shanghai 200237, China; stable_fyi@163.com (H.D.); zhaomeng03@sinochem.com (M.Z.)

**Keywords:** fluoroblock polymers, hierarchical self-assembly, phase diagram, phase behavior

## Abstract

Fluorocontaining polymers bearing special properties are unique and important materials in modern society. In this work, we focused on the phase behavior and phase diagram of poly(styrene-*block*-perfluorooctylethyl acrylate) with a volume fraction varying from 0.2 to 0.8. Small-angle X-ray scattering and transmission electron microscopy showed the phase formation in the sequence of hexagonally packed cylinders (HEX) to lamellar layers (LAM) to inverse hexagonally packed cylinders (iHEX) in this series of block polymers. Wide-angle X-ray diffraction experiments proved that the fluorodomains of the LAM phases and the matrix of iHEX phases contained layered structures formed by the crystallization of fluorosegments. During heating, the self-assembled lattice remained intact even after the melting of fluorodomain, with barely changed lattice parameters. Such hierarchical structural formation was understood by chain conformation and domain interaction, which may provide new insight into the molecular design of advanced materials.

## 1. Introduction

Fluorinated compounds possess unique aspects that are far different from those of their alkyl analogs, such as good chemical and thermal stability, low surface free energy, low fraction efficiency, biocompatibility, and nonsticky behavior [1,2,3,4,5]. However, a drawback of fluorinated polymers is that they are usually difficult to process due to their low solubility and high melting points. A feasible way to address this problem, in addition to creating new properties, is by fabricating fluorocontaining hybrid compounds in the architecture of homopolymers, block polymers, and random copolymers [6,7,8,9,10,11], or designing macromolecules bearing functional molecular nanoparticles [12,13] such as polyhedral oligomeric silsesquioxane (POSS) [14,15] and fullerene C_60_ [16]. Consequently, the immiscibility of fluorous parts with other molecules drives the self-assembly and lead to the formation of abundant mesophases and rich phase behaviors in such macromolecules [17,18,19,20]. These mesophases always show hierarchical structures that are essential for building up materials with complex responsive properties [21,22,23,24,25,26]. For example, the Ober group [17,18,27] systematically studied the relationship between self-assembled structures and critical surface tension properties of series fluorocontaining block polymers. Al-Hussein et al. [28] reported microphase segregation of poly(methyl methacrylate)-*block*-perfluorooctylethyl acrylate (PMMA-*b*-PFOA) to form cylindrical and lamellar morphologies where the perfluoroalkyl side chains intriguingly further organized into a double-layered hierarchical structure. Recently, incorporating fluorous molecular nanoparticles into polymers further showed unconventional phase structures and phase behaviors. Dong et al. [29,30] reported topological isomers made by fluorinated polyhedral oligomeric silsesquioxane (FPOSS) linked on the different positions of poly(styrene-*block*-ethylene oxide) (PS-*b*-PEO) block copolymer which showed the formation of concentric lamellae by truncated wedgelike building blocks. Hsu et al. [31] further demonstrated the thin-film self-assembly of linear/branchlike FPOSS-based surfactants to three-component perforated lamellae, cylinder within perforated lamellae, or core-shell cylinder structures. Nonetheless, the assembly of fluorocompounds in the bulk has not been fully understood due to the ill-controlled molecular parameters and the complex interplay between different driving forces for assembly.

Previously, we reported hierarchical lamellar phases formed in a series of poly(styrene-*block*-perfluorooctylethyl acrylate) (S-*b*-F) block polymers bearing perfluorooctylethyl acrylate (FOA) segments on the side-chain position, which also resemble side-chain liquid-crystal-mesogen-bearing compounds [27,32]. We further synthesized samples with a variety of volume fractions, identified the hexagonally and inversed hexagonally packed cylinder phases, and constructed a phase diagram for a thorough understanding of the self-assembly behaviors of these fluorocontaining block polymers.

## 2. Materials and Methods

Differential scanning calorimetry (DSC) was performed on a Q100 (TA Instruments, New Castle, DE, USA). Glass transition temperatures (*T_g_*) and melting temperatures (*T_m_*) were measured from the second heating profile to eliminate any influence from the thermal history. Small-angle X-ray scattering (SAXS) and wide-angle X-ray diffraction (WAXD) were recorded at Beamline 16B1 of the Shanghai synchrotron radiation facility (SSRF). The X-ray wavelength is 0.124 nm. Sample-to-detector distance was calibrated using silver behenate crystal. Transmission electron microscope (TEM) was detected by Tecnai F30 (Philips-FEI, Eindhoven, Holland) with an accelerating voltage of 120 kV. The samples were microtomed and stained by freshly prepared RuO_4_ (0.5 wt.%) vapor at room temperature for 30 min after transferring to carbon-coated copper grids.

## 3. Results and Discussion

### 3.1. General Design, Synthesis, and Phase Behavior

**Scheme 1:**
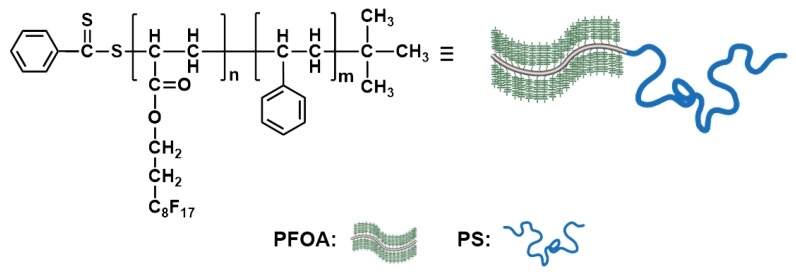
Chemical structure of poly(styrene-*block*-perfluorooctylethyl acrylate) block polymers and corresponding illustration.

**Figure 1:**
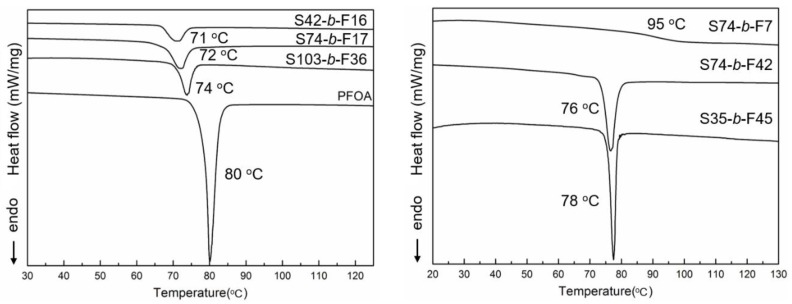
Differential scanning calorimetry (DSC) curves of S-*b*-F block polymer and perfluorooctylethyl acrylate (PFOA) homopolymer.

Detailed synthesis and characterization can be found in our previous research [33]. We first performed the reversible addition-fragmentation chain transfer polymerization (RAFT) of styrene to obtain the macroinitiators with polystyrene (PS) molecular weight varying from 3.6 to 10.7 kDa (see characterization in the supplementary materials, Figure S1, Table S1). After purification, these macroinitiators were used to perform another RAFT polymerization of FOA to obtain the desired S-*b*-F block polymers. Figures S2 and S3 show the representative ^1^H NMR (proton nuclear magnetic resonance) spectrum and gel permeation chromatography (GPC) overlay of the S-*b*-F block polymer. The polydispersity of synthesized polymers was lower than 1.25, with unimodal GPC peaks. The chemical structure of the S-*b*-F block polymers and the corresponding cartoon illustrations are shown in Scheme 1. By changing the stoichiometric ratio between initiator and monomer, we intended to cover a broad range of volume fractions that provided a phase diagram for understanding the phase behavior of fluorocontaining block polymers. All molecular weights of the synthesized block polymers were calculated on the basis of ^1^H NMR spectroscopy, and the volume fractions of poly perfluorooctylethyl acrylate (PFOA) and PS were calculated using Equations (1) and (2); density measurement was according to our previous report [33]. Results are summarized in Table 1. Glass transition temperature (*T_g_*) and melting temperature (*T_m_*) of this series of S-*b*-F block polymers were first studied using DSC under a heating/cooling rate of 10 °C/min in N_2_ atmosphere. Figure 1 shows that the melting temperatures of PFOA were molecular-weight-dependent. The homopolymer of PFOA has the highest *T_m_* of 80 °C, and the PFOA segments in block copolymers showed various degrees of melting-point depression. For the sample of S74-*b*-F7, no *T_m_* could be identified. In this case, there was a clear *T_g_* of 95 °C, which was in excellent agreement with the PS homopolymers. It was speculated that the PFOA segments were too short for crystallization in the phase-separated domain in S74-*b*-F7, whereas the glass transition of polystyrene in other samples was overwhelmed by the crystal melting of the PFOA segments. Results suggest that the block copolymers may have undergone complete phase separation.

(1) $$f_{\mathrm{PFOA}}=\frac{\left(M_{n\left(\mathrm{PFOA}\right)}/\rho_{\mathrm{PFOA}}\right)}{\left(M_{n\left(\mathrm{PFOA}\right)}/\rho_{\mathrm{PFOA}})+(M_{n\left(\mathrm{PS}\right)}/\rho_{\mathrm{PS}}\right)}$$

(2) $$\;f_{\mathrm{PS}}=\frac{\left(M_{n\left(\mathrm{PS}\right)}/\rho_{\mathrm{PS}}\right)}{\left(M_{n\left(\mathrm{PFOA}\right)}/\rho_{\mathrm{PFOA}})+(M_{n\left(\mathrm{PS}\right)}/\rho_{\mathrm{PS}}\right)}\;$$

### 3.2. Self-Assembled Morphologies and Phase Diagram

**Figure 2:**
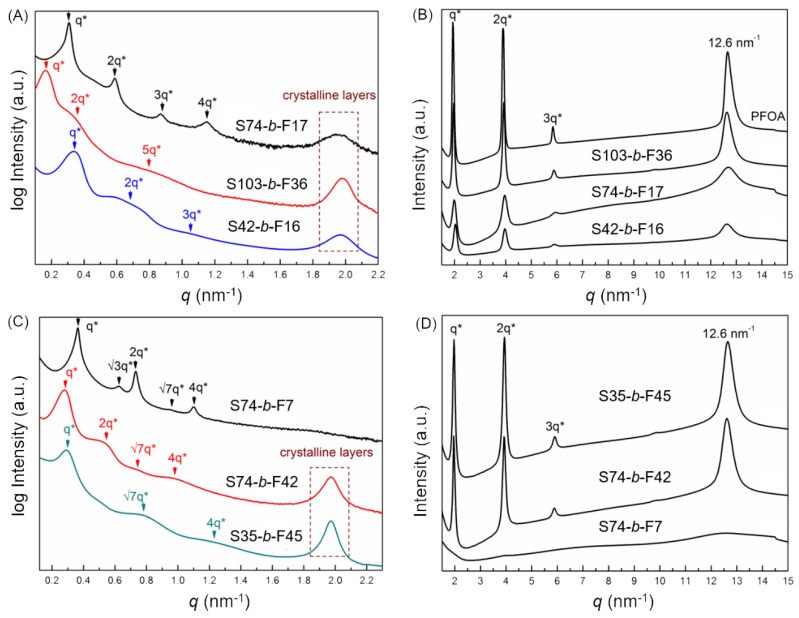
Small-angle X-ray scattering (SAXS) curves of S-*b*-F samples showing (**A**) lamellar and (**C**) hexagonal morphology. Layered crystalline PFOA structures could be identified in wide-angle region (**B**,**D**).

To probe the detailed morphologies of the different S-*b*-F block polymers, we used the SAXS and WAXD techniques. Samples were typically prepared by thermally annealing the freeze-dried powder, first at 120–140 °C (which was higher than the *T_g_* of PS and *T_m_* of PFOA segments) for 24 h to drive the phase separation, and then at 65 °C to further crystallize the PFOA segments for 24 h to fully develop the hierarchical structures. For relatively symmetric block polymers with volume fractions of PFOA (*f*_PFOA_) ranging from 0.42 to 0.55, lamellar structures were formed with various *d*-spacings (Figure 2A). When lowering *f*_PFOA_ to 0.24 or increasing to 0.64 or 0.80, hexagonally packed cylinder phases were found, as indicated by the *q* ratio of 1:√3: 2:√7: 4 for the peaks in the SAXS profile (Figure 2C). Meanwhile, the crystalline PFOA structures were characterized the diffraction peaks at ~2 nm^−1^ on the SAXS curves. We further investigated them in the wide-angle range with WAXD. As shown in Figure 2B,D, the peak ratio of 1:2:3 indicated that all samples showed a hierarchical lamellar structure except for S74-*b*-F7, where no peaks were detected in the wide-angle region consistent with the finding in DSC. From both SAXS and WAXD data, the *d*-spacing of the PFOA crystalline layer was calculated to be ~3.2 nm. This *d*-spacing was independent of the PFOA volume fraction. When PFOA was too short, as in S74-*b*-F7, crystallization was completely suppressed, which was different compared with the inversed hexagonally packed cylinders (HEX) phase in S74-*b*-F42 and S35-*b*-F45. 

**Figure 3:**
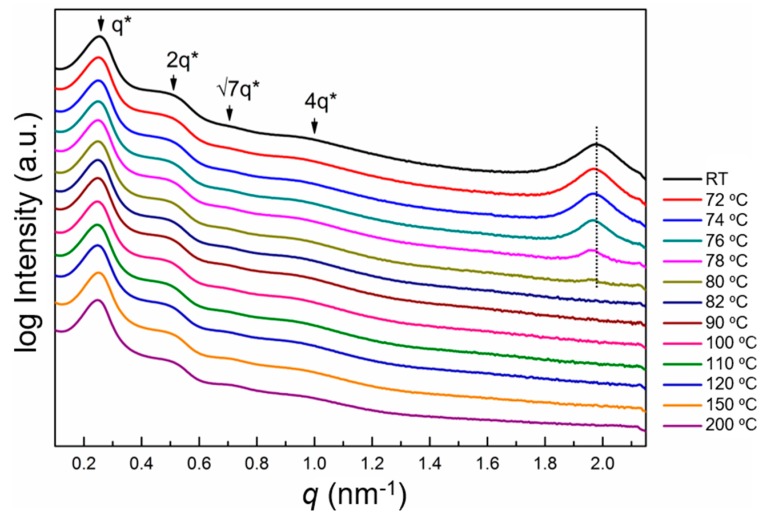
Temperature-dependent SAXS profiles of S74-*b*-F42.

**Figure 4:**
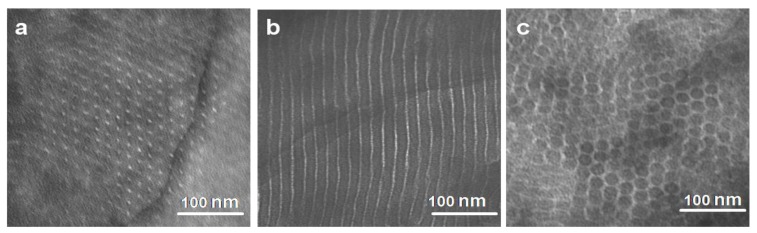
Transmission electron microscopy (TEM) micrographs of S-*b*-F block copolymers: (**a**) S74-*b*-F7, (**b**) S74-*b*-F17, and (**c**) S74-*b*-F42.

In a previous study, we inferred that PS and PFOA blocks may have high Flory–Huggins interaction parameters (*χ*), as shown by the high stability of the phase-separated lamellar structure in the temperature-dependent SAXS experiments [33]. To further demonstrate this, we performed temperature-dependent SAXS experiments on a S74-*b*-F42 sample with a hexagonally packed cylinders (HEX) phase. As shown in Figure 3, there was no change of HEX structure during heating, even up to 200 °C. This again proved that phase separation persisted throughout the heating, and the high *χ* value existed between two blocks. Results suggested that it may be useful in the pursuit of nanostructures with ultrasmall domain sizes [34]. Additionally, the melting of the PFOA segment was detected to be around 80 °C, which was consistent with DSC characterization. During the melting of PFOA, the crystalline layer expanded slightly, as indicated by the shift of the corresponding peak at ~1.9 nm^−1^. This meant that FOA segments were densely packed in the phase-separated morphology and could be treated as a hard substrate with high density [35]. Finally, the formation of lamellar and hexagonal structures was further validated in real space by observing the microtomed samples under transmission electron microscopy (TEM). Specimen were stained with RuO_4_ before imaging. Figure 4 shows the HEX, lamellar layers (LAM), and inverse hexagonally packed cylinders (iHEX) morphologies of S74-*b*-F7, S74-*b*-F17, and S74-*b*-F42, respectively. 

**Figure 5:**
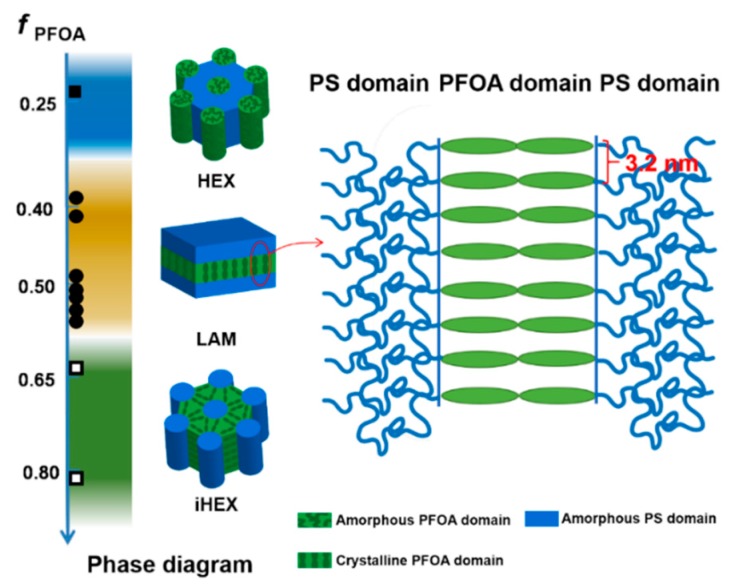
Phase diagram and illustration of hierarchical structure of S-*b*-F block copolymers. Solid square, solid sphere, and hollow square represent experiment data for hexagonally packed cylinders (HEX), lamellar layers (LAM), and inverse hexagonally packed cylinders (iHEX) phases.

The phase diagram of the S-*b*-F block copolymer was tentatively drawn in Figure 5, which only shows the LAM, HEX and iHEX phases for the experiment data. Considering that unconventional phases between LAM and HEX may only exist in a very narrow window in such strongly segregated samples [36], the results were reasonable. 

### 3.3. Hierarchical Structure and Chain Conformation

Previously, we determined the relative orientation of the PFOA crystalline layer and the phase-separated LAM structure in a S-*b*-F block copolymer by shear experiments [33]. The PFOA crystallization layers (layer spacing was 3.2 nm as detected by the WAXD experiment) were perpendicular to the phase separation layers in a LAM structure (Figure 5). Due to the chemical structure of such a side-chain liquid crystal-like PFOA, it was fair to deduce that, in the HEX phase, the crystalline PFOA layer could also lie perpendicular to the column direction [27]. Unfortunately, we failed to obtain proper data for a firm conclusion. However, different from the iHEX phase, the PFOA domain in the HEX phase of S74-*b*-F7 is noncrystalline, as shown unambiguously in the SAXS/WAXD experiments (Figure 2). 

Polymers usually undergo thermal expansion upon heating [17], and thermal shrinkage upon crystallization into densely packed states [14,37]. However, the temperature-dependent SAXS experiments of S-*b*-F block copolymers showed little shift of the ordered peaks during heating, even after melting the PFOA segments. This implied that the phase-separated structures were very stable, even in the absence of the PFOA crystallization. Hence, these nanostructured polymers could be useful as films, coatings, or other advanced materials with high thermal stability. To further shed light on such behavior of the S-*b*-F block copolymers, we calculated the stretching ratio (*S*) of the PS segments in each block copolymer. We used the radius of gyration (*R*_g_) of the unperturbed polystyrene chain as an estimate of its size in the bulk. It could be calculated using a fitted equation on the basis of experiment data from light scattering (see supplementary materials for the fitted equation) [38]. Then, *S* could be calculated by Equations (3–5), where *d* was the *d*-spacing of the first-order peak determined for the corresponding phases [39]. In the iHEX and LAM phases, the shape of the PS part was regular column and layer; thus, for Equations (3) (see supplementary materials, Figure S4, for derivation) and (4), the numerator was simply the size of PS part evaluated by the lattice dimension times *f*_PS_. In the HEX phase, the shape of the PS matrix was irregular, with different degrees of stretching. Since hexagonally packed cylinders are a 2D lattice, PS stretching was evaluated in two dimensions; thus, 2/3 of the power of the volume fraction was introduced to evaluate the average stretching [31].

(3) $$S=\frac{\sqrt{\frac{2d^{2}f_{\mathrm{PS}}}{\sqrt{3}\pi }}}{2R_{g}}\;\mathrm{for}\;\mathrm{iHEX}$$

(4) $$\;S=\frac{df_{\mathrm{PS}}}{2R_{g}}\;\mathrm{for}\;\mathrm{LAM}$$

(5) $$\;S=\frac{df_{\mathrm{PS}}^{\frac{2}{3}}}{\sqrt{3}R_{g}\left(f_{\mathrm{PS}}^{\frac{2}{3}}+f_{\mathrm{PFOA}}^{\frac{2}{3}}\right)}\;\mathrm{for}\;\mathrm{HEX}.$$

The calculated *S* values are summarized in Table 1.

The value of the interfacial area per molecule (*A*_0_) is also an important parameter to evaluate the chain conformation and stability of phase structures [40]. While it may also be calculated, however, this value is strongly influenced not only by the volume fraction and resulting morphology, but also by the molecular weights of both blocks. Thus, the comparison of *A*_0_ could no longer correctly reflect the stability of the phase. By contrast, the influence of the PS molecular weight was normalized in stretching ratio *S*. Therefore, the stretching ratio could serve as a proper indication of the conformation of PS in the assembly.

**Figure 6:**
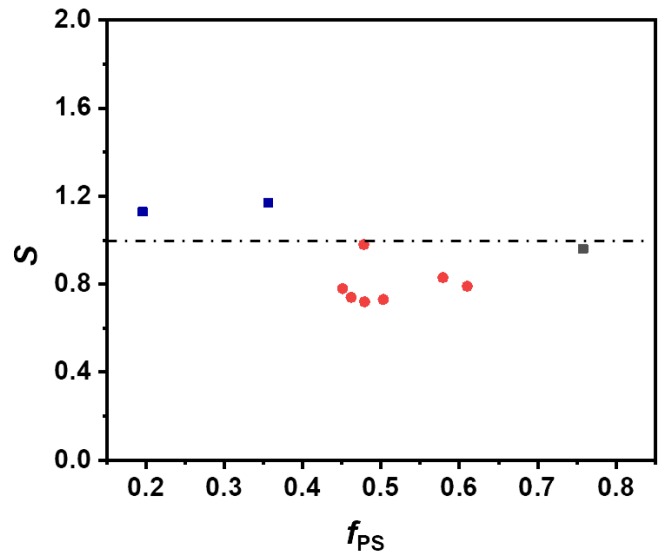
Plot of stretching ratios for polystyrene chains (*S*) versus *f*_PS_ for all S-*b*-F samples. Black square, HEX; red dot, LAM; and blue square, iHEX.

*S* values for all S-*b*-F block copolymers with different *f*_PS_ are plotted in Figure 6. Only for the iHEX phase did the PS have an *S* value larger than 1.0, meaning that the PS chain was stretched. This is consistent with the fact that PS was in the minority phase, and chains had to adopt an extended conformation stretching from the interface. For the LAM and HEX phases, the *S* values were either smaller than or not much deviated from 1.0. This means that PS chains were either compressed or relaxed. In most LAM cases, it was smaller than 1.0, which suggested that PS chains were likely compressed to a certain extent. This is probably due to the crystallization of the PFOA domain, whose extended conformation may provide more interface for the PS chains, which consequently have to compress in order to fill in space. Upon melting, the expansion of the PFOA domain in the direction perpendicular to the normal layer and the thermal expansion of the PS phase may counter each other. This may also explain the reason that *d*-spacing of the phase-separated nanostructure showed little change. 

## 4. Conclusions

In conclusion, we studied the phase behavior of a series of S-*b*-F block copolymers bearing a side-chain perfluoroalkyl segment with a volume fraction varying from about 0.2 to 0.80. The phase structures were probed using the SAXS and TEM techniques, and the phase diagram covering HEX, LAM, and iHEX phases was tentatively constructed. When the PFOA block served as the minority part within a HEX structure, the crystallization of the perfluoroalkyl chain could have been compressed to some extent. However, if the PFOA molecular weight was significantly large, the long chain still crystallized. On the other hand, when the PFOA formed the matrix in an inversed HEX phase, the crystallization of the perfluoroalkyl chain formed a hierarchical lamellar structure. Similar morphologies were also formed in the LAM structures with different volume fractions. Because such a block copolymer has high thermal stability, no thermal expansion was detected during heating. We can rationalize this phenomenon by treating the PFOA block as a dense matrix, and calculating the stretching of PS chain. Results showed that, in all cases, the PS chain was slightly stretched or compressed with a *S* value around 1.0, meaning that the conformational penalty was largely diminished under the current circumstances. Such fluorocontaining block copolymers might serve as advanced materials in future manufacturing. 

## Figures and Tables

**Table 1:** Molecular characterization of S-*b*-F block copolymers.

Sample ^a^	*f*_PS_^b^	*f*_PFOA_^b^	*M*_PS_^c^	*M*_PFOA_^d^	*q*_1_ (nm^−1^) ^e^	*d* (nm) ^f^	Structure	*R*_g_ (nm) ^g^	*S* ^h^
S74-*b*-F7	0.758	0.242	7700	3850	0.36	17.4	HEX	3.58	0.96
S74-*b*-F42	0.356	0.644	7700	21,790	0.28	22.4	iHEX	3.58	1.17
S35-*b*-F45	0.196	0.804	3600	23,310	0.29	21.2	iHEX	2.44	1.13
S74-*b*-F17	0.579	0.421	7700	8790	0.30	20.6	LAM	3.58	0.83
S42-*b*-F16	0.451	0.549	4350	8380	0.34	18.5	LAM	2.68	0.78
S103-*b*-F36	0.478	0.522	10,750	18,620	0.18	34.8	LAM	4.24	0.98
S62-*b*-F19 *	0.503	0.497	6400	10,000	0.33	19.0	LAM	3.26	0.73
S62-*b*-F21 *	0.479	0.521	6400	11,000	0.32	19.7	LAM	3.26	0.72
S91-*b*-F18 *	0.610	0.390	9450	9550	0.31	20.5	LAM	3.97	0.79
S91-*b*-F34 *	0.462	0.538	9450	17,400	0.25	25.4	LAM	3.97	0.74

^a^ Numbers in each sample represent repeating number of monomers. ^b^ Volume fractions were calculated by Equations (1) and (2), with *ρ*_PS_ = 1.05 g cm^−3^ and *ρ*_PFOA_ = 1.66 g cm^−3^. ^c^ PS molecular weights determined by GPC (gel permeation chromatography). ^d^ PFOA molecular weights determined by ^1^H NMR. ^e^ First-order peaks determined from small-angle X-ray scattering (SAXS) profiles. ^f^ Lamellar *d*-spacings calculated by *d* = 2π/*q*_1_. ^g^ PS gyration radius evaluated using Equation (S1). ^h^ Stretching ratios calculated by Equations (3–5). * Samples reported in [33]. HEX, hexagonally packed cylinders; LAM, lamellar layers; iHEX, inverse hexagonally packed cylinders.

## Supplementary Materials

The following are available online at https://www.mdpi.com/2073-4360/12/4/819/s1, Figure S1: GPC curves of PS macroinitiators., Figure S2: Representative 1H NMR spectrum of S-b-F block polymer, Figure S3: Representative GPC overlay of PS-initiator and S-b-F block polymer. Figure S4: Cartoon illustration of the iHEX phase which PS (blue part) forms the column and PFOA (green part) forms the matrix, Table S1: Molecular weight and distribution of PS macroinitiators.
