# Supplementary material for: Phase Behavior and Phase Diagram of Polystyrene-b-Poly(Perfluorooctylethyl Acrylates)
Source: Polymers (Basel). 2020 Apr 4;12(4):819. doi: 10.3390/polym12040819 (PMC7240698; doi:10.3390/polym12040819)
Supplement: Supplementary file 1 [file polymers-12-00819-s001.pdf]

Phase Behavior and Phase Diagram of Polystyrene-*b*-Poly(Perfluorooctylethyl Acrylates)

Yu Shao, Hui Dai, Meng Zhao, Bin Li, Jianan Yao, Wen-Bin Zhang and Hui Li

Chemicals and Instruments

Styrene (Shanghai Ling Feng Chemical Reagent Co., Ltd., Shanghai, China), Tetrahydrofuran (THF) (Shanghai Ling Feng Chemical Reagent Co., Ltd), and α, α, α-trifluorotoluene (TFT, Aladdin) were all purified by vacuum distillation over CaH_2 before use. Azodiisobutyronitrile (AIBN, Sinopharm Chemical Reagent Co., Ltd., Shanghai, China) was recrystallized in ethanol before use. Perfluorooctylethyl acrylates (FOA, Fuxin Heng-Tong Fluorine Chemical Co. Ltd., Liaoning, China) was washed with 5% sodium hydroxide solution to remove the polymerization inhibitor and dried over CaH_2. Benzenecarbodithioic acid 1,1-dimethylethyl ester (TTBT) was synthesized according to literature reports [1]. ^{1}H NMR spectra were recorded in $CDCl_3$/1,1,2-trichloro-1,2,2trifluoroethane (v/v, 1:1) at 500 MHz in the BRUKER Avance NMR spectrometry at 30 ± 0.2 °C. Either deuterium solvents or tetramethylsilane (TMS) served as the internal reference. Gel permeation chromatography (GPC) was performed on a PL-GPC 50 integrated GPC system (Agilent Technologies, Santa Clara, CA, USA) equipped with 2 * PLgel 5 mm MIXED-C column to obtain the molecular weights and molecular-weight distribution of the polymers at room temperature. THF was used as the mobile phase with a flow rate of 1.0 mL min^{-1} with column temperature at 30 °C, and monodisperse polystyrene standard samples were used for calibration.

Representative Molecular Characterization

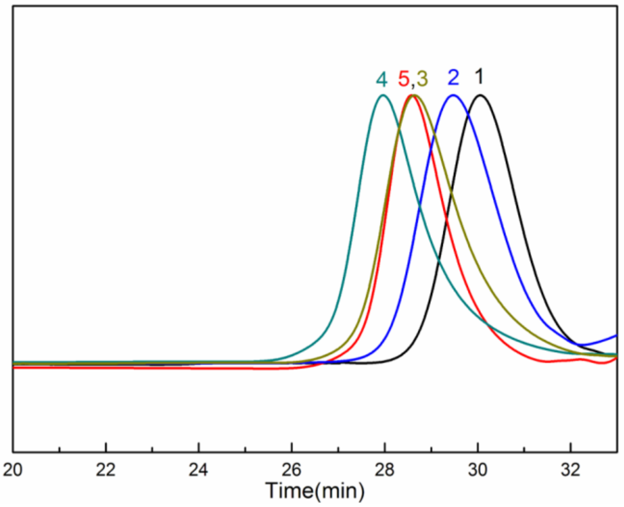

Figure S1. GPC curves of PS macroinitiators.

Table S1. Molecular weight and distribution of PS macroinitiators.

Samples	Time (h)	Conversion (%)	$M_{n(PS)}$	PDI [c]
1 [a]	3	8.6	3600	1.15
2 [a]	3.5	10.5	4350	1.16
3 [a]	5.5	19.2	8000	1.21
4 [a]	7	25.8	10750	1.21
5 [b]	24	27.1	7700	1.15

[a] [Styrene]:[initiator]:[catalyst] = 400:1:0.2. [b] [Styrene]:[initiator]:[catalyst] = 270:1:0.2. [c] The polydispersity index (PDI) is calculated by M_n/M_w obtained from GPC test.

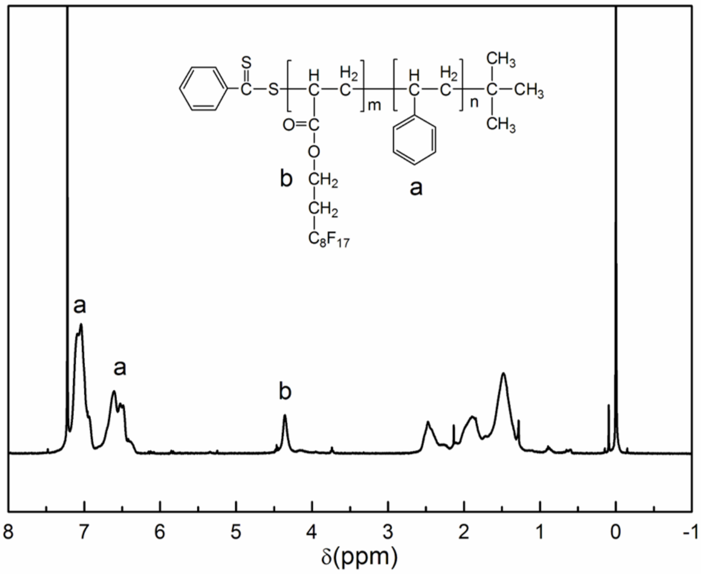

Figure S2. Representative ^{1}H NMR spectrum of S-*b*-F block polymer.

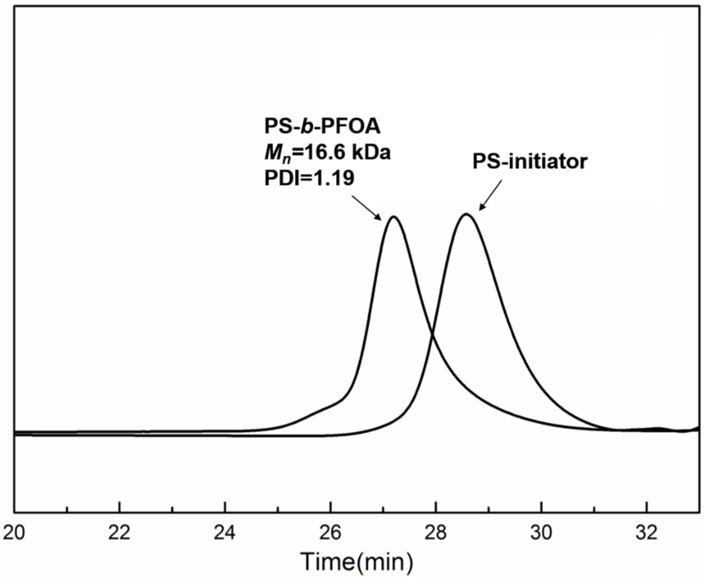

Figure S3. Representative GPC overlay of PS-initiator and S-*b*-F block polymer.

Calculation of PS Gyration Radius

Rg value was obtained from a fitted relationship between experimentally measured Rg and the molecular weight of PS as depicted in Equation (S1) [2]:

$$logR_g = 0.5063 \times logM_{n,\mathrm{PS}} - 0.3923 \quad \text{(S1)}$$

Derivation of Equation (3).

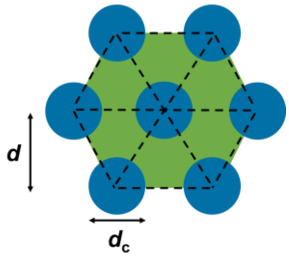

Figure S4. Cartoon illustration of the iHEX phase which PS (blue part) forms the column and PFOA (green part) forms the matrix.

$$S = \frac{\frac{d_c}{2}}{2R_g} \tag{S2}$$

Stretching ratio (S) was equal to the radius of column ($\frac{d_c}{2}$) over two times Rg.

Triangle surface area (S_t): $S_t = \frac{d^2}{\sqrt{3}}$

Total column surface area inside the dashed line hexagonal (S_c): $S_c = \frac{3\pi {d_c}^2}{4}$

Volume fraction (f_{PS}): $f_{PS} = \frac{S_c}{6S_t}$ in one unit height.

Thus, $D_c = \sqrt{\frac{8d^2 f_{PS}}{\sqrt{3}\pi}}$ lead to $S = \frac{\sqrt{\frac{2d^2 f_{PS}}{\sqrt{3}\pi}}}{2R_g}$

Reference

1. Houillot, L.; Bui, C.; Save, M.; Charleux, B.; Farcet, C.; Moire, C.; RaustIvan, J.-A.; Rodriguez, I., Synthesis of Well-Defined Polyacrylate Particle Dispersions in Organic Medium Using Simultaneous RAFT Polymerization and Self-Assembly of Block Copolymers. A Strong Influence of the Selected Thiocarbonylthio Chain Transfer Agent. *Macromolecules* **2007**, *40*, 6500–6509.
2. Wang, X.-M.; Shao, Y.; Xu, J.; Jin, X.; Shen, R.-H.; Jin, P.-F.; Shen, D.-W.; Wang, J.; Li, W.; He, J.; et al., Precision Synthesis and Distinct Assembly of Double-Chain Giant Surfactant Regioisomers. *Macromolecules* **2017**, *50*, 3943–3953.
